# Supplementary material for: Aerosolized In Vivo 3D Localization of Nose-to-Brain Nanocarrier Delivery Using Multimodality Neuroimaging in a Rat Model—Protocol Development
Source: Pharmaceutics. 2021 Mar 15;13(3):391. doi: 10.3390/pharmaceutics13030391 (PMC8001876; doi:10.3390/pharmaceutics13030391)
Supplement: Supplementary file 1 [file pharmaceutics-13-00391-s001.pdf]

pharmaceutics

Supplementary Materials: Aerosolized In Vivo 3D Localization of Nose-to-Brain Nanocarrier Delivery Using Multimodality Neuroimaging in a Rat Model—Protocol Development

Michael C. Veronesi, Brian D. Graner, Shih-Hsun Cheng, Marta Zamora, Hamideh Zarrinmayeh, Chin-Tu Chen, Sudip K. Das and Michael W. Vannier

Nanoparticles

Polylactic acid (PLA) polymer-based NPs coated with polyethylene glycoal (PEG) chains containing 20% end amino groups (PLA-PEG NPs) were synthesized (Bio Ma-Tek, Bio Materials Analysis Technology Inc., http://www.bioma-tek.com/, Hsinchu County, Taiwan). The individual compounds used to fabricate the nanoparticles are listed in Table 1. These NPs were characterized in vitro using dynamic light scattering (DLS), zeta potential measurement, and transmission electron microscopy (TEM). The NP characterizations were performed consistent with ISO 13014 [1]. The NPs were shipped dry to our laboratory in Chicago where they were reconstituted into a sterile aqueous suspension prior to experimental use in animals.

Publisher's Note: MDPI stays neutral with regard to jurisdictional claims in published maps and institutional affiliations.

Copyright: © 2021 by the authors. Submitted for possible open access publication under the terms and conditions of the Creative Commons Attribution (CC BY) license (http://creativecommons.org/licenses/by/4.0/).

Table S1. Nanoparticle Materials.

Material	Company
Ammonium thiocyanate	Sigma Aldrich
DiR (1,1'-dioctadecyl-3,3,3',3'-tetramethylindotricarbocyanine iodide), PLA [poly(D,L-latide), MW: 75,000-120,000]	Sigma-Aldrich
Iron chloride hexahydrate	Sigma-Aldrich
Chloroform	J.T. Baker
Dichloromethane	J.T. Baker
Tetrahydrofuran	J.T. Baker
DSPE-PEG2000 {1,2-distearoyl-sn-glycero-3-phosphoethanolamine-N-[methoxy (polyethylene glycol)-2000]}	Avanti Polar Lipids
DSPE-PEG2000-NH_2 {(1,2-distearoyl-sn-glycero-3-phosphoethanolamine-N-[amino (polyethylene glycol)-2000])	Avanti Polar Lipids

PLA-DSPE-PEG nanoparticle preparation

PLA-DSPE-PEG NPs were prepared as previously reported with modifications [2]. Briefly, 5 mg of PLA, 8 mg of DSPE-PEG2000 (1,2-distearoyl-sn-glycero-3-phosphoethanolamine-N-[amino(polyethylene glycol)-2000), 2 mg of DSPE-PEG2000-NH_2, and 0.1 mg of DiR were dissolved in 0.5 ml dichloromethane, and then dropped into 3 ml double-distilled water. The mixed solution was emulsified over an ice bath for 1 min using a microtip probe sonicator (XL-2000, Misonix) at 7 W output. The dichloromethane was removed by rotary evaporation to harden NPs. After centrifugation at 13,500 xg for 10 min, pellets were discarded, and NP suspension was washed three times with 10% sucrose solution by 30 kD MWCO ultrafiltration (Vivaspin 6, GE Healthcare). The purified NPs were lyophilized and stored at -20^{0}C. The materials used in nanoparticle handling and their sources are listed in Table 1. The physicochemical characteristics of these NPs were determined and are summarized in Table

Table S2. PLA-PEG NP physiochemical characterization.

Parameter	Result
1. Particle size/size distribution	
Mean diameter (TEM)	41.1 nm (n=506)
Hydrodynamic diameter (DLS)	97.1 nm
Polydispersity (DLS)	0.19
2. Aggregation/agglomeration state	No aggregation/agglomeration

3. Shape (TEM)	Fluffy sphere (negative stain)
4. Specific surface area	Not applicable
5. Composition (each eppendorf)	4.67 mg PDA01 + 50 mg sucrose
DiR	27.1 μg
DSPE-PEG2000	3.2 mg
DSPE-PEG2000-NH_2	64.7 μg (estimated)
PLA	1.41 mg
Sucrose	50 mg (estimated)
6. Surface chemistry	PEG-NH_2/PEG
7. Surface charge (zeta potential)	-36.0 mV
8. Solubility/dispersibility	≧9.3 mg PDA / ml (in 10% sucrose) Well dispersed after reconstitution

Transmission electron microscopy

Images of NPs were obtained using TEM (Hitachi model H-7650) using an acceleration potential of 100 kV. Samples were prepared by layering the nanoparticles suspension on a copper grid followed by negative staining for 10 sec with freshly prepared and sterile-filtered 2% (w/v) uranyl acetate solution. The TEM results are depicted in Fig 1 and Table 3.

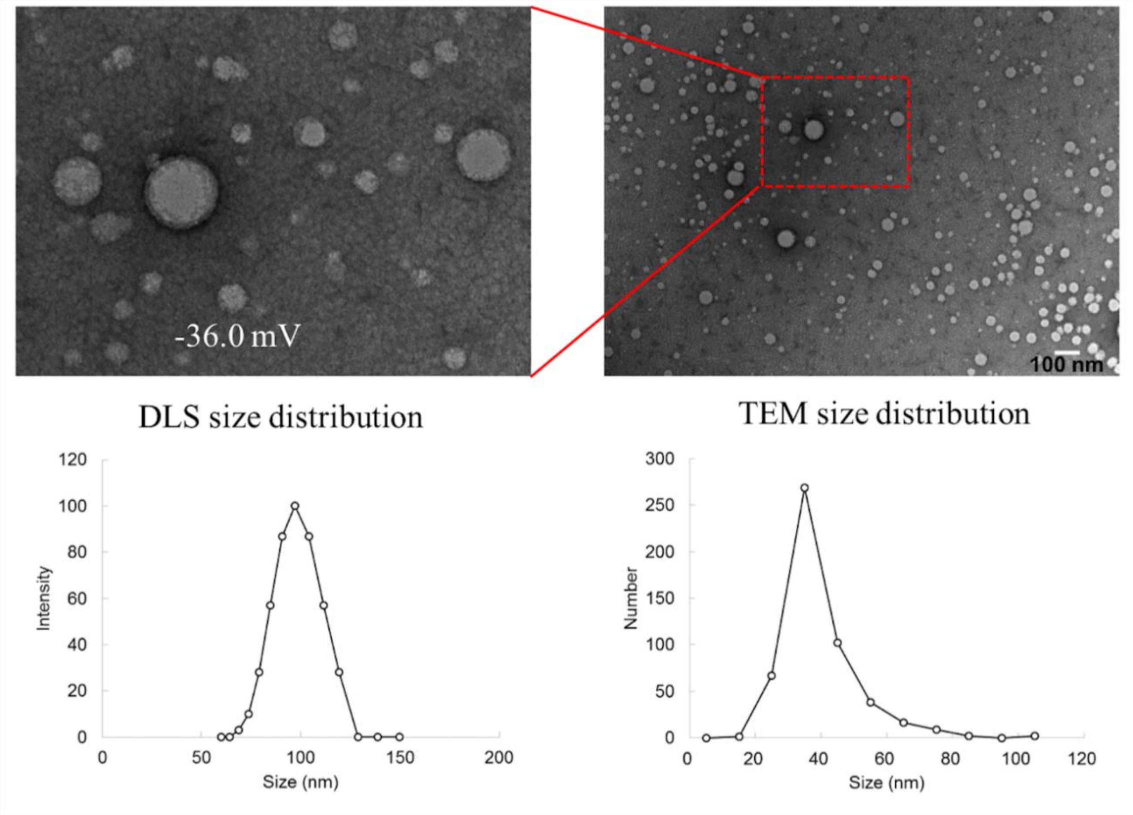

Figure S1. TEM and DLS characterization of PLA-DSPE-PEG NPs. TEM images of PLA-PEG NPs with nominal diameter up to 100 nm corresponding to the scale bar seen in the bottom right corner of the image. The particle size distribution determined by DLS and TEM are shown. The zeta potential was -36.0 mV.

Table S3. Table of Nanoparticle Properties.

System	Surfactant	Indicator	Location of indicator	Mean diameter (nm)	Zeta potential (mv)	Animal	Dose (ug)	Delivery method
Polymer-micelle (PLA-DSPE-PEG)	None	Zr89	Covalently linked (Amide linked)	50-150	-36	Sprague-Dawley	25	IN or IV

Particle size and zeta potential measurements

Hydrodynamic diameter and zeta potential of PLA-PEG nanoparticles were measured using a particle size analyzer (NanoBrook 90Plus, Brookhaven Instruments Corp., Holtsville NY) and zeta potential analyzer (NanoBrook ZetaPALS, Brookhaven Instruments Corp.) equipped with a 660-nm laser. The measured delay time correlation functions were fitted to a non-negative least squares (NNLS) model to calculate particle size distribution. The DLS results are depicted in Fig 1 and Table 3.

Quantitative determination of DSPE-PEG

DSPE-PEG was measured calorimetrically with ammonium ferrothiocyanate method [3]. Samples were dissolved in 1 ml chloroform and mixed with 1 ml of ammonium ferrothiocyanate reagent (30 mg/ml ammonium thiocyanate and 27 mg/ml iron chloride hexahydrate). The mixed solution was shaken for 3 min and centrifuged at 1,000 xg and the red lower layer was collected. The DSPE-PEG derivative was determined at 470 nm absorbance using a spectrophotometer (DU800, Beckman Coulter).

PLA quantification

Lyophilized sample was dissolved in stabilized tetrahydrofuran and 20 μl aliquot was analyzed. Chromatographic separation was performed on a gel permeation chromatography system connected to a refractive index detector (Agilent 1100 series) with a PLgel MIXED-D column (300 mm×75 mm, 5 μm, Agilent). PLA was eluted by 100% tetrahydrofuran at a 1 ml/min of flow rate and PLA content was determined by peak area of refractive index signal.

Radiolabeling of PLA-PEG NPs with ^{89}Zr.

Zirconium 89 (^{89}Zr) was produced with a cyclotron at Washington University at St. Louis and overnight shipped to our institution for nanoparticle tagging. For ^{89}Zr-labeling, PLA-PEG NPs were first conjugated with a derivative of desferrioxamine (Distearoylphosphatidylethanolamine: DFO-Bz-NCS) through amide formation. Specifically, 1.55 mg PLA-PEG NPs were stirred with 0.02 mg DFO-Bz-NCS in water for an hour. Purification (molecular weight cut-off (MWCO) 100kD) was done at 8,000 rpm with a centrifugal concentrator (Vivaspin 500 GE HealthCare) and washed with HEPES (4-(2-hydroxyethyl)-1-piperazineethanesulfonic acid) buffer. Before radiolabeling, the PLA-DSPE-PEG NPs were neutralized with ^{89}Zr-oxalate by using 1M NaOH in HEPES buffer and a final pH of 7.4 was obtained. Radiolabeled PLA-PEG NPs, ^{89}Zr (1 mCi) were added to 0.4 mg of PLA-DFO and incubated in pH 7.4 HEPES buffer for 30 minutes. Radiolabeled PLA-DSPE-PEG NPs were then purified by centrifugation and the labeling efficacy was measured with an instant thin layer chromatography (ITLC) autoradiogram. The radiolabeling activity was 650 μCi per 1 mg of PLA-PEG NPs. Details of zirconium tagging of NPs are listed in Table 4.

Table S4. Zirconium-NP tagging details.

Parameter	Result
Volume	1.2 mg NP suspended in 120 ul of 0.9% saline
Activity	967 uCi
Percent isolated yield	60% non-decay corrected yield.

References

1. International standards organization, I.T.N.-G.o.p.-c.c.o.e.n.m.f.t.a., available at: http:11.iso.org/iso/catalogue_detail?csnumber=52334 (accessed on 1 December 2020)
2. Chu, C.H.; Wang, Y.C.; Huang, H.Y.; Wu, L.C.; Yang, C.S. Ultrafine PEG-coated poly(lactic-co-glycolic acid) nanoparticles formulated by hydrophobic surfactant-assisted one-pot synthesis for biomedical applications. *Nanotechnology* **2011**, *22*, 185601, doi:10.1088/0957-4484/22/18/185601.
3. Stewart, J.C. Colorimetric determination of phospholipids with ammonium ferrothiocyanate. *Anal Biochem* **1980**, *104*, 10-14.
